# Psychological, social, and situational factors associated with COVID‐19 vaccination intentions: A study of UK key workers and non‐key workers

**DOI:** 10.1111/bjhp.12530

**Published:** 2021-05-05

**Authors:** Sarah Butter, Emily McGlinchey, Emma Berry, Cherie Armour

**Affiliations:** ^1^ Stress Trauma and Related Conditions (STARC) Research Lab School of Psychology Queen’s University Belfast Belfast Northern Ireland UK; ^2^ Centre for Improving Health Related Quality of Life (CIHRQoL) School of Psychology Queen’s University Belfast Belfast Northern Ireland UK

**Keywords:** coronavirus, COVID‐19, health behaviours, hesitancy, perceived risk, symptom severity, vaccine

## Abstract

**Objectives:**

Vaccine hesitancy is a growing concern and threat to public health. This research will begin to examine the relative influence of relevant psychological, social, and situational factors on intent to engage with a hypothetical COVID‐19 vaccine among key workers and non‐key workers.

**Design:**

Cross‐sectional.

**Methods:**

The study utilized a sample of UK adults who completed the 1‐month follow‐up of The COVID‐19 Psychological Wellbeing Study during April/May 2020 and indicated having not been previously diagnosed with COVID‐19 (key workers *n* = 584; not key workers *n* = 1,021). These groups were compared in relation to their intentions to vaccinate, perceived risk of infection, and symptom severity. Binary logistic regression was used to examine predictors of vaccine hesitancy.

**Results:**

Overall, 74.2% of the sample (76.2% key workers, 73.1% non‐key workers) indicated they would accept a COVID‐19 vaccine in future. Key workers (in particular health and social care workers) had a higher perceived risk of becoming infected in the coming months. For key workers, being female and perceiving oneself as having relatively low infection risk in the next 6 months was associated with increased likelihood of vaccine hesitancy. For non‐key workers, however, being aged 25–54, having a low or average income and not knowing someone diagnosed with COVID‐19 were associated with hesitancy.

**Conclusions:**

The proportion of individuals willing to accept a vaccine is encouraging but there is much room for improvement. Given the unique predictors of vaccine hesitancy in each group, public health campaigns may benefit from targeted messaging.

**Statement of contribution**

***What is already known on this subject?***

- Although behavioural strategies (e.g., social distancing) have been effective in limiting the transmission of COVID‐19, a longer‐term solution, such as the development of a COVID‐19 vaccine, is a global priority.
- Vaccine hesitancy (i.e., the delay in acceptance or refusal of a vaccination despite its availability) is a growing concern and threat to public health.
- Intentions to vaccinate are influenced by complex psychological, social, and situational factors, but we lack understanding of the mechanisms driving intentions to vaccinate in the unfamiliar context of COVID‐19.

***What does this study add?***

- This research provides useful early estimates on intentions to vaccinate and predictors of vaccine hesitancy.
- Unique predictors of vaccine hesitancy were found among key workers and non‐key workers, suggesting public health campaigns may benefit from targeted messaging.
- Educating the public on the extent of asymptomatic infection and transmission may be useful since most individuals believed that if infected that would exhibit some degree of symptomology.

## Background

Since late 2019, the ongoing coronavirus pandemic (COVID‐19) has rapidly swept across the globe. COVID‐19 is caused by the severe acute respiratory syndrome coronavirus 2 (SARS‐CoV‐2) which was first identified in Wuhan, China in December 2019. By 11 March 2020, the WHO had characterized the spread of COVID‐19 as a pandemic (WHO, 2020). There have now been more than 12.9 million cases and over 560,000 deaths globally, with over 312,000 cases and 43,000 deaths in the United Kingdom (as of 13 July 2020; Center for Systems Science & Engineering at John Hopkins University, 2020). Although current strategies such as social distancing, quarantine and isolation, and contact tracing have been effective in suppressing the disease, transmission will likely rebound, when these strategies are relaxed (Ferguson et al., 2020). Thus, the development of a COVID‐19 vaccine is a global priority as a longer‐term solution to overcoming this pandemic (Yamey et al., 2020).

Vaccines have been widely hailed as public health triumphs which have eradicated or greatly reduced morbidity, mortality, and health care costs associated with a range of infectious diseases (Orenstein & Ahmed, 2017). A large number of COVID‐19 vaccines are currently under development, some of which now entered the phase of human trials; however, it may be 12–18 months, at the earliest, before a COVID‐19 vaccine is approved and available for widespread use (Koirala, Joo, Khatami, Chiu, & Britton, 2020). The success of vaccines, however, does not rely solely on their availability, but also their acceptability and uptake. A high level of uptake is required to reduce incidence and prevalence of such diseases, through direct protection for vaccinated individuals and indirect protection for those who cannot be vaccinated (e.g., very young babies and those with compromised immune systems) via herd immunity (Dubé et al., 2013; Randolph & Barreiro, 2020). Although vaccination rates have been increasing for decades, recently, there has been a declining trend in some parts of Europe, including the United Kingdom (Bechini et al., 2019; NHS Digital, 2019; Nuffield Trust, 2020).

Vaccine hesitancy has been defined by the WHO Strategic Advisory Group of Experts (SAGE) on immunization as the ‘delay in acceptance or refusal of vaccination despite the availability of vaccination services. Vaccine hesitancy is complex and context specific, varying across time, place, and vaccines. It is influenced by factors such as complacency (e.g., perceived need for the vaccine), convenience (e.g., accessibility of the vaccine), and confidence (e.g., perceived benefits and safety of the vaccine)’ (MacDonald & SAGE, 2015, p. 4163). Thus, vaccine hesitancy is not confined only to those who outright reject vaccines, but those who believe that they are unsafe and therefore delay scheduled immunization programmes or those who accept some vaccinations but not others (MacDonald & SAGE, 2015; Yaqub, Castle‐Clarke, Sevdalis, & Chataway, 2014). This continuum comprises a heterogeneous group of individuals engaging in a range of vaccine‐related behaviours. Vaccine hesitancy is determined by a complex combination of contextual influences (e.g., sociodemographic factors), individual and social perceptions of vaccines (e.g., personal risk perception and knowledge), and factors related to specific vaccines (e.g., cost and mode of administration) (MacDonald & SAGE, 2015). Although previous research has examined predictors of uptake and acceptance of existing vaccines (e.g., MMR and HPV), it is important to note that the development, release, and distribution of emergency vaccines differ from that of established vaccinations (Nguyen, Holdt Henningsen, Brehaut, Hoe, & Wilson, 2011). Moreover, newer vaccines are likely to be considered less acceptable than older vaccines (Dubé et al., 2013; Larson, Cooper, Eskola, Katz, & Ratzan, 2011). Therefore, it is key that specific research is undertaken to examine acceptance of a COVID‐19 vaccination.

Perceptions of risk (i.e., perceived vulnerability of infection) and severity (i.e., severity of health consequences) are central to engagement in protective health behaviours, including adults’ intentions to vaccinate (Brewer et al., 2007; Brug, Aro, & Richardus, 2009; Fakih, Sturm & Fakih, 2020; Nguyen et al., 2011). Indeed, perceptions of risk and severity may act as important predictors of vaccination in the context of COVID‐19. Fu et al. (2020) examined COVID‐19 vaccine intentions among health care workers and the general population in China. Health care workers were more likely to believe that they might be infected in future and were more willing to accept a vaccine (76.4% compared to 72.5% of the general public). Among the determinants of vaccine acceptance in health care workers and the public was high possibility of infection (30%+), with these individuals being around twice as likely to accept a vaccine. Disease severity was not associated with vaccination intentions in health care workers but was very weakly associated in the general population, unexpectedly exhibiting a negative trend. Such psychological factors extend to perceptions of safety and effectiveness in a newly developed vaccine. For example, Faasse and Newby (2020) studied predictors of health protective behaviours, including vaccination intentions among the Australian public. If a safe and effective COVID‐19 vaccine was developed, 81.1% reported that they ‘definitely would’ or ‘probably would’ accept it, while 19.0% were reported being ‘unsure’ or that they 'probably' or 'definitely' would not get it. This study found that along with a number of sociodemographic and COVID‐19 related variables, likelihood of infection was marginally associated with increased intentions to get vaccinated whereas disease severity was not.

For many individuals, perceived risk of being infected may be influenced by their ability to stay at home and limit their contact with others. In the United Kingdom, the government defined a range of critical workers or ‘key workers’, whose jobs are essential and, as such, these workers were encouraged to continue to carry out work during lockdown period, while others with non‐essential jobs were furloughed or asked to work from home. Defined key worker roles include those working in health and social care, education, transport, key public services, local or national government, food and necessity goods, public safety, and certain utilities, communications, and financial services (UK Government, 2020). Key workers represent a unique group of individuals within COVID‐19 society. For non‐key workers, lockdown measures have resulted in limited contact with others, limited travel from home and either working from home or being furloughed. Key workers, however, due to the essential nature of their jobs, have not had this experience. In many cases, key worker roles cannot be conducted from home. These individuals, particularly health care workers (and their families), are at greater risk infection due to high‐risk working environments (e.g., hospitals), increased contact outside of the home and potential inability to partake in health protective behaviours, for example, due to lack of personal protective equipment or inability to social distance (The Lancet, 2020a, 2020b). Indeed, frontline health care workers are at an increased risk of COVID‐19 infection (Nguyen et al., 2020). However, key workers comprise of more than health care workers, and outbreaks and increased risk among food processing and transport staff, for example, have also been documented (Office for National Statistics [ONS], 2020a; The Lancet, 2020b).

With vaccine acceptance largely determining the success of a prospective COVID‐19 vaccine, this study aims to investigate the anticipated uptake of a COVID‐19 vaccine, should it become available in the future, among key workers and non‐key workers in the UK, and importantly also, to begin explore the mechanisms underpinning anticipated COVID‐19 vaccine hesitancy. Previous research affirms the complexity of vaccine hesitancy and suggests that intentions to take a novel COVID‐19 vaccine will be determined by a range of factors (Brewer et al., 2007; Brug et al., 2009; Fakih, Sturm, & Fakih, 2020; Nguyen et al., 2011; WHO, 2019a). As captured by the Increasing Vaccination Model (Brewer, Chapman, Rothman, Leask, & Kempe, 2017; WHO, 2019a), intentions to vaccinate (generally) are influenced by complex psychological, social, and situational factors, but we lack understanding of the mechanisms driving intentions to vaccinate in the unfamiliar context of COVID‐19. Thus, this research will begin to examine the relative influence of relevant psychological (perceived risk and severity), social, (media exposure), and situational (demographic and medical) factors on intent to engage with a hypothetical COVID‐19 vaccine. Moreover, given the important circumstantial distinctions between key workers and non‐key workers, this study will investigate predictors of hesitancy towards a COVID‐19 vaccine separately across each group.

## Methods

### Sample

The current study is based on a cross‐sectional sample of UK adults who took part in the 1‐month follow‐up survey of The COVID‐19 Psychological Wellbeing Study. Participants were recruited via social media platforms (e.g., Twitter and Facebook) and the online participant panel *Prolific*. Participants completed an online baseline survey between 23 March and 24 April 2020. All respondents were prompted to complete a 1‐month follow‐up survey 30 days after completing their baseline survey, which included information on vaccines and risk perceptions. All 1‐month follow‐ups were completed online between 22 April and 18 May 2020.^1^ In total, 1,660 valid cases completed the 1‐month follow‐up survey (83.4% of baseline cases). For the current study, only individuals who reported not having been previously diagnosed with COVID‐19 (formally diagnosed, diagnosed by GP, or self‐diagnosed) were included (*N* = 1,605). Of these, 584 (36.4%) worked in Government‐assigned key worker roles. The remainder of the sample (*n = *1,021, 63.6%) were not key workers. Detailed information on the survey development, measures, procedures, and overall baseline sample characteristics can be found at Armour, McGlinchey, Butter, McAloney‐Kocaman and McPherson (2021).

### Ethical approval

Ethical approval was granted by the Queen’s University Belfast’s Engineering and Physical Sciences Faculty Research Ethics Committee. All participants provided informed consent prior to completing the questionnaire.

### Measures

#### Vaccination intentions

At 1‐month follow‐up, participants’ willingness to accept a COVID‐19 vaccine themselves, should it become available in future, was assessed. Specifically, this item asked ‘If a new vaccine was to be developed for coronavirus (COVID‐19) and was available to you, would you accept it for yourself?' Responses were coded as ‘No’ (0), ‘Yes’ (1), and ‘Don’t know’ (2). Given that vaccine hesitancy comprises delay in acceptance as well as refusal, both ‘no’ (refusal) and ‘don’t know’ (uncertainty) responses were considered to fall within the vaccine hesitancy spectrum.

#### Sociodemographic characteristics

Sociodemographic information was collected on all participants in the baseline survey.

- *Age:* Age was grouped into five possible categories: 18–24, 25–34, 35–44, 45–54, 55+ years (coded 1–5). Due to few respondents aged 65+ within the original sample, this age bracket was combined with age 55–64 years for the purposes of the current study.
- *Gender:* Respondents were asked to identify their gender, this was recoded as male = 0, female = 1. Due to few respondents endorsing any other gender categorization, ‘other’ responses were recoded and treated as missing data for the purpose of this analysis.
- *Income:* Respondents were asked to rate their income as ‘less than average’ (0), ‘average’ (1) or ‘more than average’ (2).
- *Education:* A variable assessing educational achievement identified respondents whose highest educational attainment was a qualification at secondary level or less (0), having a post‐secondary level qualification up to and including at undergraduate degree level (1) and having a postgraduate degree qualification (2). Endorsement of ‘other’ level of education was recoded and treated as missing.
- *Urbanicity:* Respondents were asked ‘What type of area do you live in?’. Responses to this item were recoded to living in a rural area (1), a town (2), or a city (3).
- *Children:* Respondents were asked how many children they had. Responses were collapsed to indicate whether they had a child/children (1) or did not have any children (0).
- *Country:* Respondents were asked what country within the UK they resided in: Northern Ireland (1), Scotland (2), or England/Wales (3). England and Wales were combined in the current study due to the low frequency of Welsh respondents in the sample.
- *Pre‐existing physical and mental health condition:* This variable categorized whether respondents had ever suffered from a physical health condition (inclusive of asthma, heart disease, cancer, diabetes, shortness of breath, and self‐reported other chronic physical health condition) and if they had ever suffered from a mental health condition (inclusive of post‐traumatic stress disorder, major depressive disorder, phobia, social phobia, obsessive compulsive disorder, generalized anxiety disorder, psychosis, eating disorder, health anxiety, and self‐reported other chronic mental health condition). These variables were coded yes (1) and no (0).

#### COVID‐19 variables (measured at 1‐month follow‐up)

- *COVID‐19 risk:* Respondents were asked what they believed their risk of getting COVID‐19 was in the next month, 3 months, and 6 months. Responses were rated on scale from 0 to 100%.
- *COVID‐19 symptom severity:* Respondents were asked if they were to get COVID‐19 what severity of symptoms they believed they would experience. This was rated on 5‐point Likert scale ranging from asymptomatic/no symptoms (1) to deadly symptoms (5).
- *COVID‐19 exposure:* Respondents were asked whether, at the time of completing the survey, they knew someone who was currently or in the past had been diagnosed with COVID‐19. This variable was coded as yes (1), no (0). Responses of ‘don’t know’ were recoded and treated as missing data.
- *COVID‐19 media consumption:* Frequency of watching, reading, and hearing reports or updates about COVID‐19 on social media (e.g., Twitter, Facebook, and WhatsApp) and on traditional media (e.g., TV, radio, and newspaper) over the past month were assessed. Responses were dichotomized to indicate ‘low exposure’ (1; 0 – 5 times a day) and ‘high exposure’ (2; 6 or more times a day).

### Analytic plan

Firstly, vaccination intentions, perceived COVID‐19 risk, and perceived symptom severity were examined among the full sample and compared by key worker status using chi‐squares, Fisher’s exact test, and independent samples *t*‐tests. Next, two separate binary logistic regression analyses were conducted predicting vaccination hesitancy (i.e., ‘no or ‘don’t know’ responses) among key workers and non‐key workers separately.

## Results

### Sample characteristics

Sociodemographic characteristics of the sample are presented in Table 1. The key worker sample (*n* = 584) was made up of those working in health and social care (26.9%), education and childcare (24.3%), transport (3.4%), key public services (7.7%), local or national government (9.6%), food and other necessity goods (12.2%), public safety (3.4%), and utilities, communication and financial services (12.3%).

**Table 1:** Sociodemographic and COVID‐19‐related sample characteristics

	*N* (%)	
	Key workers (*n* = 584)	Non‐key workers (*n* = 1,021)
Gender		
Male	146 (25.0)	347 (34.3)
Female	437 (75.0)	664 (65.7)
Age		
18–24	60 (10.3)	211 (20.7)
25–34	197 (33.7)	320 (31.3)
35–44	175 (30.0)	210 (20.6)
45–54	100 (17.1)	136 (13.3)
55+	52 (8.9)	144 (14.1)
Area		
Rural	128 (21.9)	208 (20.4)
Town	274 (46.9)	455 (44.6)
City	182 (31.2)	358 (35.1)
Education		
Full secondary or less	132 (22.7)	316 (31.2)
Post‐secondary including undergraduate degree	294 (50.5)	479 (47.3)
Postgraduate qualification	156 (26.8)	218 (21.5)
Income		
Below average	144 (24.7)	475 (46.5)
Average	322 (55.1)	410 (40.2)
Above average	118 (20.2)	136 (13.3)
Children		
Yes	303 (51.9)	442 (43.3)
No	281 (48.1)	579 (56.7)
Country		
Northern Ireland	140 (24.0)	206 (20.2)
Scotland	202 (34.6)	341 (33.4)
England/Wales	242 (41.4)	474 (46.4)
Physical health condition		
Yes	177 (30.3)	321 (31.4)
No	407 (69.7)	700 (68.6)
Mental health condition		
Yes	148 (25.3)	350 (34.3)
No	436 (74.7)	671 (65.7)
Social media exposure		
Low	452 (78.1)	803 (78.6)
High	127 (21.9)	217 (21.3)
Traditional media exposure		
Low	486 (83.9)	885 (86.7)
High	93 (16.1)	135 (13.2)
Know someone diagnosed		
Yes	290 (50.7)	363 (36.7)
No	282 (49.3)	627 (63.3)

Overall, approximately three‐quarters (74.2%) of the sample reported that they would accept a vaccine, while 17.7% were uncertain and 8.1% reported that they would refuse it (Table 2). A chi‐square test of association revealed that the association between vaccination intentions and key worker status was non‐significant. Perceived risk was examined in relation to the next month, 3 months, and 6 months. In the total sample, the average perceived risk of being infected with COVID‐19 in the next month was 32% which increased to 42% within the next 6 months. Independent samples t‐test found that on average, key worker risk perceptions were higher than non‐key worker in each of the cases. Finally, regarding perceived symptom severity, most individuals believed that they would have mild (41%) or moderate (40%) symptomology. A minority believed that symptoms would be serious for them (12%) while few individuals thought that they would experience no symptoms (5%) or deadly symptoms (2%). A chi‐square test of association revealed no association between perceived symptom severity and key worker status.

**Table 2:** Vaccination intentions, perceived risk of contracting COVID‐19, and perceived symptom severity among key workers and non‐key workers

	Total sample	Key ;workers	Non‐ key workers	χ^2^ (df), *p*
Vaccine intentions (*N* = 1,599)	Count (%)			
Acceptance (‘Yes’)	1187 (74.2)	441 (76.2)	746 (73.1)	5.91 (2), *p* > .05
Refusal (‘No’)	129 (8.1)	34 (5.9)	95 (9.3)	
Uncertainty (‘Don’t know’)	283 (17.7)	104 (18.0)	179 (17.5)	

Perceived COVID‐19 risk (*N* = 1,602)	*M*(*SD*)			*t*‐test
…in next month	32.36 (22.02)	37.66 (22.07)	29.34 (21.43)	*t*(1600) = −7.39, *p* < .001
…in next 3 months	38.60 (23.56)	43.27 (22.88)	35.95 (23.54)	*t*(1600) = −6.05, *p* < .001
…in next 6 months	41.52 (27.10)	45.10 (27.11)	39.47 (26.89)	*t*(1600) = −4.02, *p* < .001

Perceived symptom severity (*N* = 1,602)	Count (%)			χ^2^ (df), *p*
Asymptomatic	79 (4.9)	30 (5.2)	49 (4.8)	1.73 (4), *p* > .05
Mild	656 (40.9)	230 (39.6)	426 (41.7)	
Moderate	641 (40.0)	243 (41.8)	398 (39.0)	
Serious	197 (12.3)	67 (11.5)	130 (12.7)	
Deadly	29 (1.8)	11 (1.9)	18 (1.8)	

As there were significant differences in perceived risk of infection between key workers and non‐key workers, follow‐up comparisons were made between key workers in health and social care (HSC) and all other key workers (Table 3). There were no significant differences between HSC key workers and other key workers on vaccination intentions or perceived symptom severity. However, HSC workers had higher perceived risk of infection within the next month and the next 3 months. There was no significant difference between the groups regrading risk of infection within the next 6 months.

**Table 3:** Vaccination intentions, perceived risk of contracting COVID‐19, and perceived symptom severity among health and social care key workers and other key workers

	Total key worker sample	HSC workers	Other key workers	χ^2^ (df), *p*
Vaccine intentions (*N* = 579)	Count (%)			
Acceptance (‘Yes’)	441 (76.2)	117 (75.5)	324 (76.4)	4.34 (2), *p* > .05
Refusal (‘No’)	34 (5.9)	14 (9.0)	20 (4.7)	
Uncertainty (‘Don’t know’)	104 (18.0)	24 (15.5)	80 (18.9)	

Perceived COVID‐19 risk (*N* = 581)	*M*(*SD*)			*t*‐test
…in next month	37.66 (22.07)	41.26 (21.23)	36.35 (22.24)	*t*(579) = −2.39, *p* < .05
…in next 3 months	43.27 (22.88)	46.69 (23.55)	42.02 (22.53)	*t*(579) = −2.19, *p* < .05
…in next 6 months	45.10 (27.11)	47.86 (27.84)	44.09 (26.80)	*t*(579) = −1.49, *p* >.05

Perceived symptom severity (*N* = 581)	Count (%)			Fisher’s Exact test, *p*
Asymptomatic	30 (5.2)	6 (3.8)	24 (5.6)	4.82, *p* > .05
Mild	230 (39.6)	66 (42.3)	164 (38.6)	
Moderate	243 (41.8)	70 (44.9)	173 (40.7)	
Serious	67 (11.5)	13 (8.3)	54 (12.7)	
Deadly	11 (1.9)	1 (0.6)	10 (2.4)	

Note

HSC Health and Social Care. Fisher’s exact test used due to low cell counts (<5).

Predictors of vaccine hesitancy, compared to acceptance, were examined separately in both groups (Table 4). Given the small proportion of individuals perceiving themselves to exhibit no symptoms or deadly symptoms if infected, these response options were combined with mild symptoms and serious symptoms responses, respectively, for inclusion in the regression model. Furthermore, risk perceptions were categorized into four groups representing low perceived risk of infection through to very high risk of infection.

**Table 4:** Binary logistic regression analyses predicting vaccine hesitancy in key workers (*N* = 565) and non‐key workers (*N* = 972)

Predictors	OR (CI 95%)	
	Key workers (*N* = 565)	Non‐key workers (*N* = 972)
Gender		
Male		
Female	1.96 (1.16–3.32)*	1.15 (0.83**–**1.59)
Age		
18–24	–	–
25–34	1.77 (0.81**–**3.89)	2.41 (1.48–3.94)***
35–44	2.07 (0.89**–**4.80)	1.96 (1.12–3.45)*
45–54	1.99 (0.79**–**5.02)	2.91 (1.62–5.24)***
55+	1.56 (0.52**–**4.63)	1.81 (0.95**–**3.45)
Area		
Rural		
Town	0.77 (0.45**–**1.31)	1.10 (0.73**–**1.66)
City	0.90 (0.50**–**1.60)	1.13 (0.73 −1.75)
Education		
Secondary or below	–	–
Post‐secondary	0.97 (0.57**–**1.64)	1.04 (0.73**–**1.48)
Postgraduate	1.06 (0.57**–**1.99)	0.79 (0.50**–**1.25)
Income		
Above average	–	–
Average	1.28 (0.72**–**2.26)	2.37 (1.34–4.19)**
Below average	1.43 (0.73**–**2.77)	2.58 (1.45–4.60)***
Has child/children		
No	–	–
Yes	0.75 (0.46**–**1.20)	1.17 (0.82**–**1.67)
Country		
England/Wales	–	–
Northern Ireland	1.05 (0.63**–**1.76)	0.75 (0.47**–**1.07)
Scotland	0.63 (0.39**–**1.02)	0.85 (0.60**–**1.21)
Physical health condition		
No	–	–
Yes	1.18 (0.74**–**1.88)	1.07 (0.76**–**1.51)
Mental health condition		
No	–	–
Yes	0.95 (0.59**–**1.52)	0.98 (0.70**–**1.35)
COVID‐19 social media exposure		
Low	–	–
High	0.85 (0.48**–**1.49)	0.98 (0.66**–**1.45)
COVID‐19 traditional media exposure		
Low	–	–
High	0.72 (0.37**–**1.39)	0.76 (0.47**–**1.23)
Know someone diagnosed		
No	–	–
Yes	0.80 (0.53**–**1.21)	0.61 (0.44–0.84)**
Perceived symptom severity		
Serious/deadly	–	–
Moderate	1.14 (0.58**–**2.25)	1.34 (0.81**–**2.24)
None/Mild	1.21 (0.59**–**2.47)	1.45 (0.85**–**2.46)
6‐month risk		
75–100%	–	–
51–75%	1.44 (0.69**–**3.03)	0.95 (0.54**–**1.69)
26–50%	1.92 (0.97−3.81)	0.87 (0.52**–**1.46)
0–25%	2.44 (1.22–4.91)*	1.49 (0.91**–**2.46)

Notes

Significant odds ratios (ORs) in bold.

* *p *< .05

** *p *< .01

*** *p *< .001.

In the key worker sample, only two characteristics were associated with vaccine hesitancy: being female (compared to male; *OR* = 1.96) and perceiving oneself as having a relatively low risk (0–25%) of being infected with COVID‐19 in the next 6 months (compared to very high perceived risk: 75–100%; *OR* = 2.44). In the non‐key worker sample, several factors were associated with vaccine hesitancy: being aged 25–34 (*OR* = 2.41), 35–44 (*OR* = 1.96), and 45–54 (*OR* = 2.91) compared to 18–24 year olds, having an average (*OR* = 2.37) or below average income (*OR* = 2.58) compared to an above average income. Additionally, knowing someone who is diagnosed with COVID‐19 was associated with reduced risk of vaccine hesitancy (*OR* = 0.61).

## Discussion

The current study examined intentions to engage with a prospective COVID‐19 vaccine and the psychological, social, and situational factors influencing vaccine hesitancy in a UK sample of key workers and non‐key workers in the two months following UK lockdown. The Increasing Vaccination Model (Brewer et al., 2017; WHO, 2019a) provided a useful scaffolding to guide this exploratory work. Specifically, the influence of perceptions of risk of infection and symptom severity, media exposure (as a key feature of social environment during lockdown), and sociodemographic variables on vaccination intentions was measured in both groups. The findings suggest that the psychological and social factors influencing vaccination intentions differ across the vocational groups under study. This has important implications for the nuances of vaccine hesitancy and the theory underpinning this complex phenomenon, because it highlights the importance of context. With vaccine hesitancy listed as one of the top ten threats to global health in 2019 (WHO, 2019b), and in light of the ongoing pandemic, understanding the mechanisms driving hesitancy and identifying groups who are most likely to be hesitant is paramount. Moreover, with greater understanding of this, public health information, campaigns, or interventions can be contextualized and targeted towards those most susceptible to vaccine hesitancy.

### Vaccination intentions, perceived risk of infection, and symptom severity

Overall, 74.2% of the sample indicated that they would accept a COVID‐19 vaccine if one was developed in future and there was no significant association between key worker status and intentions to vaccinate. These rates are comparable to what has been previously reported in the United Kingdom and China (69.0 and 75.0%, respectively; Fu et al., 2020; Murphy et al., 2021). It is slightly higher than reported among an Irish sample (65%; Murphy et al., 2021) and slightly lower than that in an Australian sample (81% said that they would definitely or probably accept a vaccine; Faasse & Newby, 2020). For COVID‐19, thresholds for herd immunity (i.e., the proportion of immune individuals needed to halt the spread of infection) has been estimated to lie between 50% and 75%, although higher estimates have also been proposed (up to 85%) dependent on the region being examined (Kwok, Lai, Wei, Wong, & Tang, 2020). Therefore, the proportion of individuals intending to get vaccinated in this study is encouraging, yet there is clearly room for improvement. Importantly, however, these figures only reflect *intentions* to vaccinate, which may vary from actual *behaviour* (Ajzen, 1991; Sheeran & Webb, 2016). This is especially of relevance since, at the time of the study, no vaccines were available, and therefore, this situation was hypothetical. Once vaccines become available to the public and specific details regarding its cost, effectiveness, etc. are known, these intentions may need to be re‐evaluated.

UK key workers believed that they were more at risk of being infected in the coming months compared to non‐key workers. Similar perceptions have been reported among health care workers in other countries (Fu et al., 2020; Peres, Monteiro, Almedia, & Ladeira, 2020). Furthermore, this aligns with monitoring statistics which suggest that health care workers, along with those in other key working roles such as factory and transport workers, have been most affected by COVID‐19 (Mutambudzi et al., 2021; Nguyen et al., 2020; ONS, 2020a). Indeed, in this study, HSC key workers had a greater average perceived risk of infection than other key workers regarding the next month and next 3 months.

Additionally, most respondents (~80%) believed that they would experience mild or moderate symptoms if infected, while few believed that they would be asymptomatic or experience deadly symptoms. As noted by Faasse and Newby (2020), there is a worrying implication of the low proportion of individuals who perceived that they would experience asymptomatic COVID‐19. Although there are still many uncertainties and unknowns on the subject, recent reviews suggest that the rate of asymptomatic cases may be between 15% and 81% (Byambasuren et al., 2020; Ing, Cocks & Green, 2020; Oran & Topol, 2020). Asymptomatic individuals are also believed to be able to transmit the virus and a ‘silent spread’ of the virus has been attributed to such cases (Huang, Li, Dunn, & He, 2021; Oran & Topol, 2020). If the majority of people expect, perhaps incorrectly, that they will exhibit symptomology if infected with the disease, this may motivate asymptomatic individuals to fail to comply with health protective behaviours (e.g., social distancing). Campaigns should aim to educate the public about the prevalence of asymptomatic infection and transmission, highlighting the need to remain vigilant and continue to engage in health protective behaviours (e.g., social distancing) even in the absence of symptoms.

### Predictors of vaccine hesitancy

Sociodemographic and COVID‐19‐related variables uniquely predicted vaccine hesitancy in the key worker and non‐key worker samples in this study. Perception of likely symptom severity did not significantly predict vaccination intentions in either group, as has previously been reported (Faasse & Newby, 2020), however, perceived risk of infection was associated in the key worker sample only. Thus, risk perceptions alone may not be sufficient enough to motivate adoption of health protective behaviours (Brug et al., 2009; Fakih et al., 2020; West, Michie, Rubin, & Amlôt, 2020). For example, high‐risk perceptions may only predict behaviour when people believe that effective protective actions are available and when they believe that they have the ability to engage in such actions (Brug et al., 2009; Fakih et al., 2020). As many of the key workers in this sample work in health and social care, it may be possible that they are more likely to believe that vaccinations are safe and effective protective behaviours, and as such, their perceptions of risk motivate them to intend to vaccinate. Risk perceptions are also influenced by communication of risk via government agencies and the media for example (Fakih et al., 2020) and misinformation can contribute to harmful, inaccurate beliefs about vaccines (Smith, 2017).

In this study, there was no association with social or traditional media exposure to COVID‐19 information. However, these variables captured *frequency* of COVID‐19 media exposure rather than *content*. In future investigations, it may be valuable to explore the relative influence of other psychological factors such as emotions (e.g., fear and disgust), perceived control and self‐efficacy, and social norms/expectations in the context of a COVID‐19 vaccine to establish a more comprehensive understanding of intentions to vaccinate. Consideration of a broader range of psychological and social variables aligns with the Increasing Vaccination Model (Brewer et al., 2017; WHO, 2019a), and the current findings can usefully guide this future work. The findings also demonstrate that, while theoretical models provide scaffolding to explore the psychological and social factors influencing vaccine hesitancy, there are important differences across the two vocational populations in relation to the factors driving vaccine hesitancy. This is understandable given the differences in level of physical and psychological exposure to COVID‐19. This bears relevance for theory as it prompts us to acknowledge the role of contextual factors (i.e., vocational position) and affirms the need for targeted interventions that are sensitive to contextual differences. Specifically, interventions targeting vaccine hesitancy should be tailored to address the unique barriers experienced by those in key working roles and those who work outside this domain.

The significant sociodemographic predictors (age, gender, and income) in this study corroborate other recent COVID‐19 and previous vaccine acceptance research (e.g., Faasse et al., 2020; Murphy et al., 2021), as well as some research on established vaccines, for example, MMR, influenza (Bish, Yardley, Nicoll & Michie, 2011; Sandford, Tata, Browne, & Pritchard, 2015). Understanding the characteristics of individuals that are reluctant to accept emergency pandemic vaccines may inform public health strategies to promote vaccination uptake and plan ahead in anticipation of the development of vaccines and their rollout (Nguyen et al., 2011). Public health campaigns may benefit from targeting public health messaging most relevant to each group of reluctant individuals. Key workers may particularly benefit from targeting towards females, who make up the majority of the key working population and are most likely to work in health and caring professions with vulnerable individuals (ONS, 2020b). Communicating their increased risk of infection status, due to increased interaction with the others outside of the home and highlighting the social responsibility to act in a manner to protect vulnerable others, even if they themselves are at low risk, may also be beneficial. Non‐key worker, general public campaigns may benefit from targeting middle‐aged adults, those on low‐to‐average incomes and who do not know any individuals diagnosed with COVID‐19. Local authorities may consider educating about the impact of COVID‐19 in their community specifically to highlight the extent of infection, spread, and severity.

### Limitations

Several limitations of this study should be noted. Although uncertainty (‘don’t know’) and refusal (‘no’) were combined in this study to examine hesitancy, since they are considered to both fall within the definition of hesitancy as proposed by the WHO and others (e.g., Dubé et al., 2013; MacDonald & SAGE, 2015), it is important to note that there are differences between these responses. Vaccine attitudes can be considered as a continuum of attitudes ranging from ‘active demand’ to ‘complete refusal’, with uncertainty located somewhere in the middle (Dubé et al., 2013), and furthermore, uncertainty may precede outright refusal (Yaqub et al., 2014). Indeed, this definition of hesitancy has been criticized as ambiguous (Peretti‐Watel, Larson, Ward, Schulz, & Verger, 2015). Thus, there are important distinctions to be drawn between these groups that may be useful to study in further detail, particularly since there may be more scope to change the attitudes of those who are uncertain than those who outright refuse. Indeed, recent research from the United Kingdom and Ireland has reported both a number of similarities and differences in the characteristics of those who were uncertain about whether they would accept a COVID‐19 vaccine and those who were resistant (Murphy et al., 2021).

Furthermore, given that the results of the current study are cross‐sectional and collected during the early stages of the pandemic, continual monitoring of vaccination intentions among the general population, as well as specific subgroups, will be necessary as the pandemic rapidly evolves, and as vaccination schedules are implemented. Unfortunately, this study did not have access to information on other variables which are known important predictors of vaccination intentions, such as perceived vaccine effectiveness and safety, knowledge, and trust in the health care community (Dubé et al., 2013; Larson et al., 2011; Yaqub et al., 2014). These underlying attitudes are of utmost importance and should be considered in future research into intentions to vaccinate against COVID‐19. Such studies would also benefit from the inclusion of more robust, psychometrically assessed COVID‐19 and vaccination‐related measures, if available.

Additionally, the sample in this study was not representative of the general population of the United Kingdom, and therefore, these findings may not be generalizable to the population as a whole. Particular groups such as males, older adults (65+), and lower levels of education were underrepresented. A detailed description of the sample characteristics and their comparison to UK‐wide demographic can be found elsewhere (Armour et al., 2021). It is also important to acknowledge that the definition of key worker used in this study, although in line with the Government‐defined categories, is broad. There is potential that some respondents who indicated being employed in a key worker role may have been furloughed by their employer who chose to operate business using a reduced staff numbers, others may be able to carry out their essential duties at home, while some may be vulnerable and shielding due to health conditions. This may have affected their perceived risk of infection, symptom severity, and intentions to vaccinate. Generally however, although heterogeneous, individual in key worker roles, due to the nature of their jobs, may have less opportunity to social distance and have more interactions outside of the home, both at their workplace and on public transport.

### Conclusion

This study suggests that around three‐quarter of the population report intentions to accept a COVID‐19 vaccine if it became available to them. Key workers and non‐key workers exhibited unique characteristics predictive of vaccine hesitancy. Gender and perceptions of risk of infection were associated with key workers while age, income, and not knowing someone diagnosed with the disease were associated with non‐key workers. As such, public health campaigns may wish to target their messaging based on these characteristics. By doing so, if a COVID‐19 vaccination is successfully developed, uptake rates may be increased, the impact on vulnerable individuals reduced and a healthy population maintained. Further research will be needed as the COVID‐19 situation evolves and if a vaccine is developed. However, this research provides useful early estimates and guidance from a public health standpoint.

## Conflicts of interest

All authors declare no conflict of interest.

## Author contributions

Sarah Butter (Conceptualization; Formal analysis; Methodology; Writing – original draft; Writing – review & editing) Emily McGlinchey (Conceptualization; Investigation; Methodology; Writing – review & editing) Emma Berry (Writing – review & editing) Cherie Armour (Conceptualization; Investigation; Methodology; Writing – review & editing.

## Data Availability

Participants did not provide consent for their data to be made publicly available; however, we will provide the relevant code and output files derived from the analyses. The raw data corresponding to the paper may be made available upon reasonable request to the principal investigator (Armour) in conjunction with an appropriate data sharing agreement.

## Footnotes

^1^
At the initiation of this phase of data collection (22 April 2020), the total number of laboratory confirmed positive COVID‐19 cases in the United Kingdom was 140,202 and the total number of deaths within 28 days of a positive test result was 23,582 (Public Health England, 2020; https://coronavirus.data.gov.uk/).
